# Neurochemical and functional interactions for improved perceptual decisions through training

**DOI:** 10.1152/jn.00308.2021

**Published:** 2022-03-02

**Authors:** Ke Jia, Polytimi Frangou, Vasilis M. Karlaftis, Joseph J. Ziminski, Joseph Giorgio, Reuben Rideaux, Elisa Zamboni, Victoria Hodgson, Uzay Emir, Zoe Kourtzi

**Affiliations:** ^1^Department of Psychology, University of Cambridge, Cambridge, United Kingdom; ^2^Purdue University School of Health Sciences, West Lafayette, Indiana

**Keywords:** functional connectivity, learning, MR spectroscopy, perceptual decisions, transcranial direct current stimulation

## Abstract

Learning and experience are known to improve our ability to make perceptual decisions. Yet, our understanding of the brain mechanisms that support improved perceptual decisions through training remains limited. Here, we test the neurochemical and functional interactions that support learning for perceptual decisions in the context of an orientation identification task. Using magnetic resonance spectroscopy (MRS), we measure neurotransmitters (i.e., glutamate, GABA) that are known to be involved in visual processing and learning in sensory [early visual cortex (EV)] and decision-related [dorsolateral prefrontal cortex (DLPFC)] brain regions. Using resting-state functional magnetic resonance imaging (rs-fMRI), we test for functional interactions between these regions that relate to decision processes. We demonstrate that training improves perceptual judgments (i.e., orientation identification), as indicated by faster rates of evidence accumulation after training. These learning-dependent changes in decision processes relate to lower EV glutamate levels and EV-DLPFC connectivity, suggesting that glutamatergic excitation and functional interactions between visual and dorsolateral prefrontal cortex facilitate perceptual decisions. Further, anodal transcranial direct current stimulation (tDCS) in EV impairs learning, suggesting a direct link between visual cortex excitation and perceptual decisions. Our findings advance our understanding of the role of learning in perceptual decision making, suggesting that glutamatergic excitation for efficient sensory processing and functional interactions between sensory and decision-related regions support improved perceptual decisions.

**NEW & NOTEWORTHY** Combining multimodal brain imaging [magnetic resonance spectroscopy (MRS), functional connectivity] with interventions [transcranial direct current stimulation (tDCS)], we demonstrate that glutamatergic excitation and functional interactions between sensory (visual) and decision-related (dorsolateral prefrontal cortex) areas support our ability to optimize perceptual decisions through training.

## Graphical abstract

**Figure:**
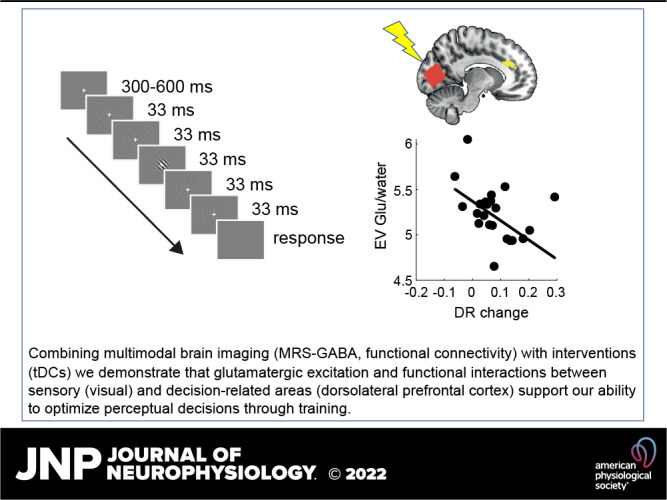

## INTRODUCTION

Making successful perceptual judgments entails integrating multiple sources of sensory information over time (1, 2). For example, when deciding whether we have spotted a friend in a crowd, we accumulate information over time (e.g., as they approach, their appearance, clothing, and gait become clearer) and take into account not only the immediate sensory input but also our previous experience and knowledge (e.g., the likelihood of them appearing there and then).

Computational investigations have advanced our understanding of perceptual decision making by using sequential sampling models to decompose behavioral responses into decision processes (3, 4). In these sequential sampling models, participants accumulate evidence for two alternative choices and make their response when a critical amount of information (i.e., decision threshold) has been obtained in favor of one choice over the other. Previous work has implicated a network of regions in evidence accumulation for perceptual decision making, including parietal (5), frontal (6), prefrontal (7, 8), and ventral premotor (9) cortex.

Further, previous behavioral (10–12) and neuroimaging (13–15) studies have proposed a role of learning in perceptual decision making, showing that training enhances evidence accumulation for perceptual judgments (e.g., discrimination of visual features) (11, 14, 16–18). Yet, our understanding of the brain mechanisms that alter decision processes through training remains limited.

Here, we interrogate the neurochemical and functional brain mechanisms that support our ability to improve our perceptual decisions because of training. We focus on perceptual learning, that is, our ability to improve our perceptual judgments with training. We used an orientation identification task that involves identifying the orientation of a Gabor grating from Gaussian noise (19). We modeled behavioral performance using the drift diffusion model (i.e., a widely used sequential sampling model) (3, 4) to identify the decision processes involved in orientation identification and test the effect of training on these processes, rather than overall task performance.

Visual perceptual learning has been shown to engage a network of visual regions involved in sensory processing and frontoparietal regions involved in decision making (for reviews see Refs. 20, 21). In particular, training has been shown to alter processing in both visual cortex (22–25) and higher frontoparietal areas (14, 15, 26). Here we focus on early visual cortex (EV) and the dorsolateral prefrontal cortex (DLPFC), which is known to be functionally connected to EV cortex (27) and involved in perceptual decision making (7, 8).

Further, previous studies have investigated the role of excitatory [glutamate (Glu)] and inhibitory [γ-aminobutyric acid (GABA)] neurotransmitters in visual processing and learning. Thanks to recent advances in magnetic resonance spectroscopy (MRS), it is now possible to reliably measure these neurotransmitters noninvasively in the human brain. MRS studies have shown that glutamatergic excitation, which is known to play a key role in long-term potentiation induction and plasticity (for a review see Ref. 28), relates to visual cortex activation (29, 30), contrast sensitivity (31), motion discrimination (32), and object recognition (33). GABAergic inhibition in the visual cortex, as measured by MRS, has been implicated in orientation discrimination tasks (34–36) and visual perceptual learning (37–39). Further, the neurochemical balance between excitation and inhibition has been suggested to play a key role in brainwide network interactions (40). Human MRS studies have linked Glu and GABA concentrations at rest with functional connectivity as measured by resting-state functional magnetic resonance imaging (rs-fMRI) (41–44), consistent with the role of glutamatergic excitation and GABAergic inhibition in neural dynamics.

Here, we ask whether neurochemical processing within visual and decision-related areas and functional interactions between these regions relate to improved perceptual decisions due to training. Using MRS to measure neurotransmitter levels at rest, we test whether Glu and GABA (referred to as GABA+to account for coedited macromolecules) levels in EV and DLPFC relate to learning-dependent changes in decision processes. Using rs-fMRI, we test whether functional connectivity between these regions relates to glutamatergic excitation or GABAergic inhibition and learning-dependent changes in decision processes. Our results demonstrate that training on an orientation identification task enhances information accumulation (i.e., improved drift rate). This behavioral improvement relates to lower EV glutamatergic excitation and functional connectivity between EV and DLPFC, suggesting that local excitatory processing in visual cortex and interactions between visual and decision-related areas contribute to optimizing perceptual decisions through training. Moving beyond correlational evidence, we use transcranial direct current stimulation (tDCS) to perturb cortical excitability during training on the orientation identification task. Our results show that increasing excitation with anodal stimulation of the early visual cortex impairs learning in the orientation identification task, suggesting that low levels of excitation in the visual cortex are directly linked to efficient sensory processing for improved perceptual decisions.

## MATERIALS AND METHODS

### Participants

Twenty-five participants (12 female, 13 male; mean age 24 ± 3.7 yr) took part in the main study, and forty participants (13 female, 27 male; age 21 ± 2.3 yr) took part in the tDCS experiment (20 in the Anodal group and 20 in the Sham group). All participants were right-handed, had normal or corrected-to-normal vision, did not receive any prescription medication, and gave written informed consent. The study was approved by the University of Cambridge Research Ethics committee (PRE.2017.57).

### Experimental Design

Participants in the main study took part in one behavioral session in the laboratory and two brain imaging scans (before behavioral training) comprising rs-fMRI and MRS. Participants in the tDCS study took part in one behavioral session with stimulation in the laboratory.

### Stimuli and Task

Experiments were controlled with MATLAB and Psychophysics Toolbox 3.0 (45, 46). For the behavioral session, stimuli were presented on a 21-in. CRT monitor (1,600 × 1,200-pixel resolution, 60-Hz frame rate) with gamma correction at a distance of 50 cm. Stimuli comprised oriented Gabor patches that were presented against a uniform gray background. Gabor patches of random phase had a fixed diameter of 12°, SD of the Gaussian envelope of 2°, contrast of 0.03, and spatial frequency of 1 cycle/°. Gaussian-distributed noise patterns had a contrast of 0.2. This contrast value was defined based on a pilot study that showed 60% accuracy in orientation identification before training. An assessor independent from the researchers who ran the experiments monitored the pretraining performance during data collection. The first set of eight participants of the Anodal group were tested on the same contrast level as participants in the main experiment (i.e., 0.03). However, they showed lower pretraining accuracy than the expected 60% (mean accuracy = 55.9%). Therefore, we increased the contrast of the stimuli to 0.035 for the remaining participants in the tDCS experiment (12 for Anodal, 20 for Sham). Statistical analyses with and without the participants who performed the task with lower contrast showed similar results.

**Figure 1.**
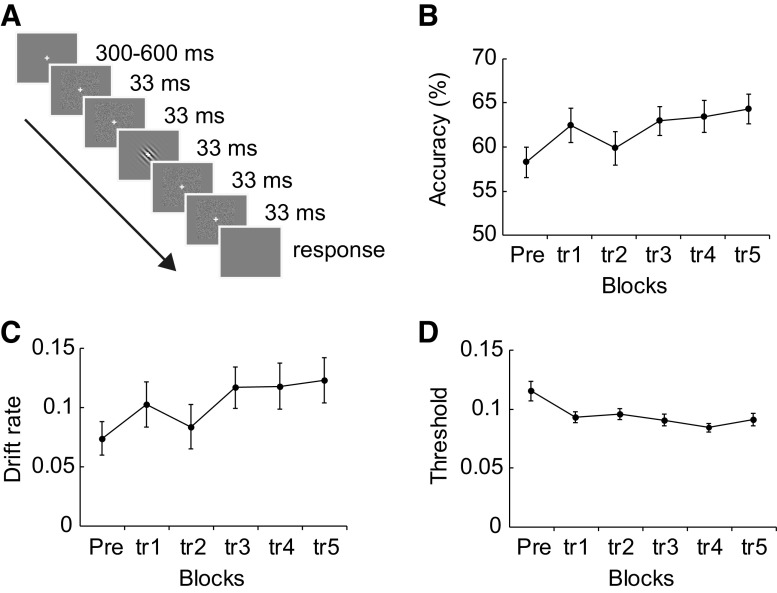
Behavioral task and performance. *A*: orientation identification task. Participants judged the orientation of a Gabor patch presented (45° or 135°) among Gaussian noise patterns. *B*: mean performance across participants for the pretest (Pre) and training (tr1–tr5) blocks. *C* and *D*: mean drift rate (*C*) and threshold (*D*) derived from diffusion modeling [drift rate, decision threshold, nondecision time (DR-TH-Ter) model] across participants for the pretest and training blocks. Error bars indicate SE across participants. We used the Bayesian information criterion (BIC) for 5 constructed models and selected the DR-TH-Ter model with the lower BIC value (i.e., null model: 3,246.22, DR model: 3,267.02, DR-TH model: 3,234.13, DR-TH-Ter model: 3,228.73, full model: 3,308.36).

We tested participants on an orientation identification task (Fig. 1*A*) during a test block (100 trials; no feedback) followed by five training blocks (100 trials each, with per-trial feedback). Each trial began with a fixation cross for a jittered duration between 300 and 600 ms (in increments of 100 ms) followed by the noise patterns and Gabor patches. Two Gabor frames (i.e., 33 ms) were presented in between pairs of noise frames (i.e., 4 noise frames were presented before and after the Gabor frames) to ensure temporal integration of the Gabor and noise patterns (19). Participants were asked to fixate and judge the orientation (left vs. right) of the Gabor patch (45° or 135°; Fig. 1*A*).

### MRI Data Acquisition

We collected MRI data on a 3-T Siemens PRISMA scanner (Wolfson Brain Imaging Unit, Cambridge) using a 32-channel head coil. We acquired longitudinal relaxation time (T1)-weighted (T1w) structural data [magnetization-prepared rapid gradient echo (MPRAGE); repetition time (TR) = 2 s; echo time (TE) = 2.98 ms; number of slices = 176; voxel size = 1 mm isotropic) and echo-planar imaging (EPI) data at rest (gradient echo-pulse sequences; TR = 0.727 s; TE = 34.6 ms; number of slices = 72; voxel size = 2 mm isotropic; multiband factor = 8; flip angle = 48°; number of volumes = 825; duration = 10 min; whole brain coverage). EPI data comprised two runs (10 min per run), during which participants fixated on a cross in the middle of the screen.

**Figure 2.**
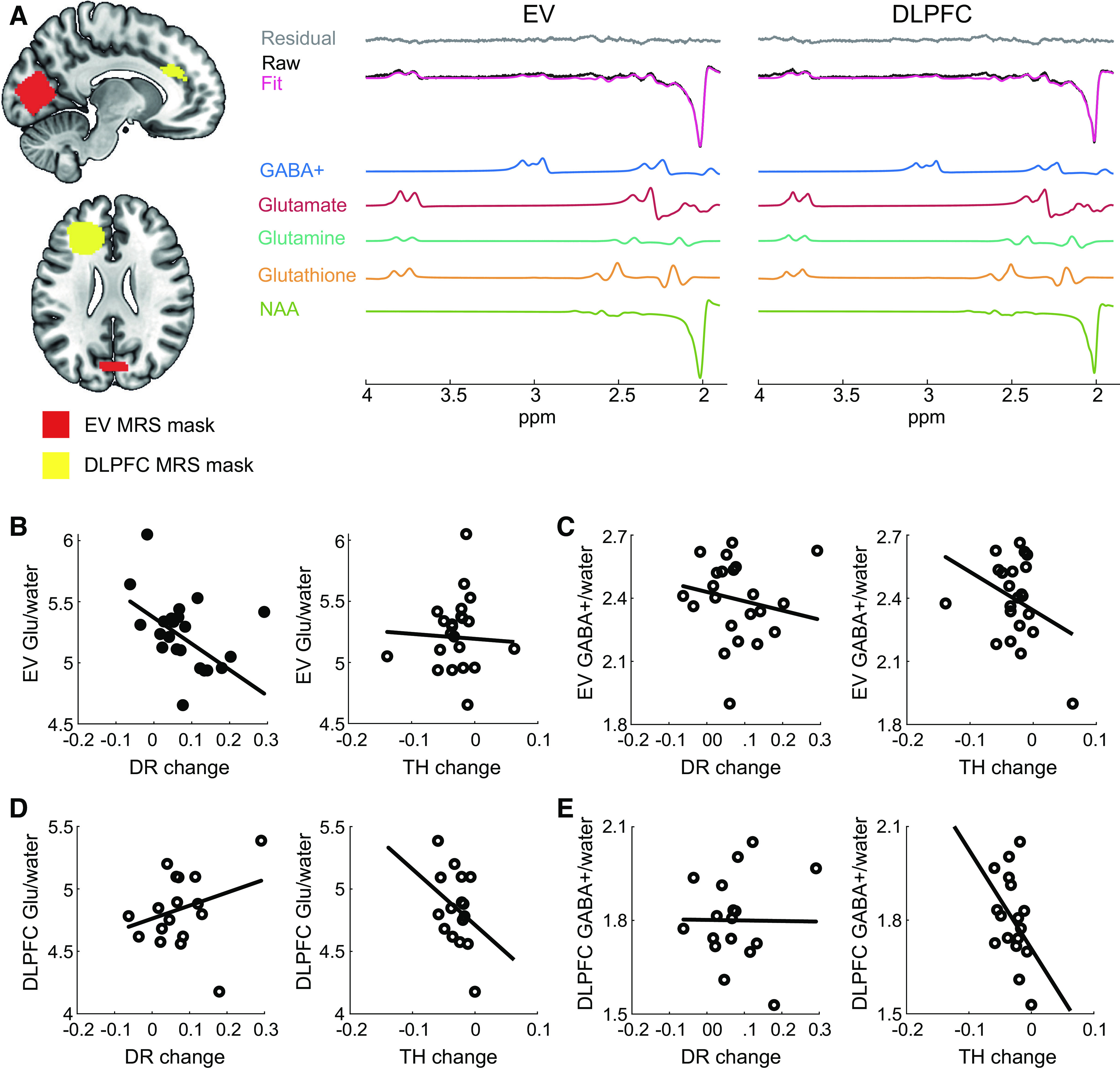
Relationship of magnetic resonance spectroscopy (MRS) glutamate and GABA+ to behavior. *A*: MRS voxels and spectra in the early visual cortex (EV) and dorsolateral prefrontal cortex (DLPFC). We illustrate a group MRS mask (sagittal, axial view) that covers a cortical area that is common in at least 50% of the participants’ MRS voxels (red, EV; yellow, DLPFC). Sample spectra from the MRS voxels show the LCModel fit, residual, and respective fits for GABA+, glutamate (Glu), glutamine, glutathione, and *N*-acetylaspartate (NAA). *B*: multiple regression of EV Glu with behavior: significant negative linear relationship with drift rate (DR) but not decision threshold (TH) change (max-training block minus pretraining block). *C*: no significant linear relationship of EV GABA+ with behavior. *D*: no significant linear relationship of DLPFC Glu with behavior. *E*: no significant linear relationship of DLPFC GABA+ with behavior. Significant results are indicated by filled symbols and nonsignificant results by open symbols.

We collected MRS data with a 32-channel head coil and a MEGA-PRESS sequence (47): TE = 68 ms; TR = 3,000 ms; 256 transients of 2,048 data points were acquired in 13-min experiment time; a 14.28-ms Gaussian editing pulse was applied at 1.9 (ON) and 7.5 (OFF) ppm; water unsuppressed 16 transients [Supplemental Table S1 (all Supplemental Material is available at https://doi.org/10.17863/CAM.82236), following consensus guidelines (48)]. Water suppression was achieved by using variable power with optimized relaxation delays and outer volume suppression. We conducted automated shimming followed by manual shimming. We acquired spectra from two MRS voxels (25 × 25 × 25 mm^3^): in early visual cortex (EV voxel) and the left dorsolateral prefrontal cortex (DLPFC voxel) (Fig. 2*A*). We manually positioned the MRS voxels using anatomical landmarks on each participant’s T1 scan, ensuring that voxel placement was consistent across participants. The EV voxel was placed medially in the occipital lobe with the lower face aligned with the cerebellar tentorium and as posterior as possible toward the occipital pole given the voxel dimensions. The DLPFC voxel was placed within the left hemisphere and above the superior margin of the lateral ventricles. The center of gravity for the EV voxel was *x* = 0.8 ± 1.8 mm, *y* = −80.2 ± 2.4 mm, *z* = 8.2 ± 2.9 mm in MNI space (partially covering V1 and V2 regions) and for the DLPFC voxel was *x* = −24.4 ± 2.0 mm, *y* = 33.0 ± 7.0 mm, *z* = 25.1 ± 6.2 mm in MNI space. The order of the voxels was counterbalanced across participants. During the MRS acquisitions participants fixated on a cross in the middle of the screen to encourage similar levels of alertness across participants.

### tDCS Data Acquisition

We used a multichannel transcranial electrical stimulator (neuroConn DC-STIMULATOR MC, Ilmenau, Germany) to deliver anodal or sham stimulation in a double-blind manner. We used a pair of rubber electrodes (3 × 3-cm^2^ stimulating electrode, 5 × 5-cm^2^ reference electrode), placed in square sponges that had soaked in saline. For anodal stimulation, 1-mA current was ramped up over 10 s, held at 1 mA for the duration of training (∼25 min), and subsequently ramped down over 10 s. For sham stimulation, the current ramped up (10 s) and down (10 s) in the beginning of the session. To achieve consistent electrode placement across participants when targeting the early visual cortex, we used a 10-20 System EEG cap as reference and centered the anode on Oz and the cathode on Cz. This montage has been extensively used in tDCS studies targeting the early visual cortex (e.g., Refs. 49, 50) and has been shown to successfully increase excitability in this region (51).

### Data Analysis

#### Behavioral data analysis.

Three participants from the main study and one from the tDCS experiment (from the Sham group) were excluded because of high starting performance (over 75%). We further excluded seven participants from the tDCS experiment (2 from the Anodal group and 5 from the Sham group) because of atypical response times (RTs) (i.e., RT < 0.2 s) that suggested the participants did not engage with the task. This resulted in *N* = 22 for the main study and *N* = 32 for the tDCS experiment (*N* = 18 for Anodal, *N* = 14 for Sham), consistent with sample sizes in our previous studies (37). After previous studies using a single training session (38) we calculated performance accuracy per participant and compared accuracy in the pretraining block to accuracy in the max-training block (i.e., we selected the block with the higher accuracy between the last 2 training blocks per participant to account for potential fatigue effects toward the end of the training).

Further, to model processes related to decision making, we fitted the behavioral data for each block with the Diffusion Model Analysis Toolbox (DMAT; Refs. 52, 53). The drift diffusion model (DDM) consisted of seven parameters: *1*) The mean drift rate (DR) and *2*) across-trial variability (*s*) in drift rate indicate stimulus discriminability; that is, a higher drift rate denotes faster and more accurate responses. The drift rate varies from trial to trial, following a normal distribution with mean DR and standard deviation *s*. *3*) The decision threshold (TH) controls the speed-accuracy trade-off and represents the amount of evidence required for making a decision. A higher decision threshold denotes slower but more accurate responses, suggesting that participants tend to make more cautious decisions. *4*) The mean starting point (z) and *5*) variability of starting point (sz) reflect the observer’s prior bias at stimulus onset. In the case of the diffusion model, the starting point of the decision process at stimulus onset is assumed to vary randomly from trial to trial, according to a uniform distribution with mean *z* and standard deviation sz. This random variation may reflect, for example, the influence of recent preceding trials. *6*) The mean nondecision time (Ter) and *7*) variability of nondecision time (st) denote the time that includes early encoding processes (i.e., before the diffusion decision process) and late motor response processes (i.e., after the diffusion decision process). The nondecision time is assumed to vary randomly across trials according to a uniform distribution with mean Ter and standard deviation st. The diffusion model assumes that the observed response time is the sum of the nondecision component and the diffusion decision component.

Based on previous studies (11, 12), we constructed five different models. *Model 1* assumed that learning did not change any parameter of the model (null model); *model 2* assumed that learning changed drift rate (DR model); *model 3* assumed that learning changed drift rate and decision threshold (DR-TH model); *model 4* assumed that learning changed drift rate, decision threshold, and nondecision time (DR-TH-Ter model); *model 5* assumed that learning changed all the parameters of the model (full model). We used the Bayesian information criterion (BIC) for the five constructed models and selected model DR-TH-Ter that had the lowest mean BIC value across participants (i.e., null model: 3,246.22, DR model: 3,267.02, DR-TH model: 3,234.13, DR-TH-Ter model: 3,228.73, full model: 3,308.36). Quantile-probability plots were used to inspect the model fitting. Data from one participant in the tDCS study (from the Anodal group) were excluded from further analysis as the model fit did not converge.

#### MRS data analysis.

We preprocessed the MRS data with MRspa v1.5c (www.cmrr.umn.edu/downloads/mrspa/). We applied eddy current, frequency, and phase correction before subtracting the average ON and OFF spectra, resulting in edited spectra. We used LCModel (54) to quantify metabolite concentrations by fitting model spectra of glutamate (Glu), glutamine (Gln), γ-aminobutyric acid (GABA), glutathione (GSH), and *N*-acetylaspartate (NAA) to the edited spectra (Fig. 2*A*). The model spectra of all metabolites were generated based on previously reported chemical shifts and coupling constants using the GAMMA/PyGAMMA simulation library of VESPA (Versatile Simulation, Pulses and Analysis, Ref. 55) for carrying out the density matrix formalism. A 20 × 20 spatial matrix was used to simulate the spatial variations inside and outside the nominal PRESS dimensions. Simulations were performed with the same radio frequency (RF) pulses and sequence timings used on our 3-T scanner.

We focused on glutamate rather than glutamine, as it is the primary excitatory neurotransmitter and it is known to play a key role in brain plasticity and learning (56). Glutamate has been shown to be separable from glutamine and reliably quantified when measured with MEGA-PRESS at 3 T (57) and the spectra are fitted using LCModel (58, 59). Our glutamate measurements are in agreement with the spectral quality criteria outlined in previous work (57). Following these criteria, we were able to distinguish glutamate from glutamine for most participants. We conducted additional control analyses, excluding data in cases that Gln could not be fit (*n* = 4, EV voxel).

We refer to GABA concentration as “GABA+,” as MRS measurements of GABA with MEGA-PRESS include coedited macromolecules (60). We referenced Glu and GABA+ concentrations to the concentration of water and validated our findings by referencing Glu and GABA+ to NAA to ensure that our results were not driven by the chosen reference (61).

All spectra had linewidth below 10 Hz and Glu and GABA+ Cramér–Rao lower bound (CRLB) values smaller than 10%. DLPFC data for five participants were excluded because of lipid contamination, as detected by visual inspection by two independent reviewers (P. Frangou, J. J. Ziminski), resulting in *N* = 22 for EV and *N* = 17 for DLPFC. Signal-to-noise ratio (SNR) was computed with LCModel as the amplitude of the NAA peak in the difference spectrum divided by twice the root mean square of the residual signal (54). We report average concentrations of Glu and GABA+, in addition to quality indexes (CRLB, linewidth, SNR), per MRS voxel (Supplemental Table S2). To control for potential differences in data quality across participants, we performed control analyses that accounted for variability in absolute CRLB (62), linewidth, and SNR across participants.

Further, we conducted whole brain tissue type segmentation of the T1-weighted structural scan and calculated the percentage of gray matter (GM), white matter (WM), and cerebrospinal fluid (CSF) voxels in each MRS voxel. We then divided the Glu and GABA+ concentrations by [1 − CSF fraction] to ensure that our results were not driven by variability in tissue composition within the MRS voxel across participants and used these tissue-corrected values in further analyses.

#### rs-fMRI data preprocessing.

We preprocessed the rs-fMRI data in SPM12.3 (v6906; www.fil.ion.ucl.ac.uk/spm/software/spm12/), following the Human Connectome Project (HCP) pipeline for multiband data (63). In particular, we first coregistered (nonlinear) the T1w structural images (after brain extraction) to MNI space to ensure that all participant data were in the same stereotactic space for statistical analysis. We then *1*) corrected the EPI data for any spatial misalignments between EPI volumes due to head movement (i.e., aligned each run to its single-band reference image), *2*) coregistered the second EPI run to the first run (rigid body) to correct any spatial misalignments between runs, *3*) coregistered the first EPI run to the structural image (rigid body), and *4*) normalized them to MNI space for subsequent statistical analyses (applying the deformation field of the structural images). Data were only resliced after MNI normalization to minimize the number of interpolation steps. After MNI normalization, data were *5*) skull-stripped, *6*) spatially smoothed with a 4-mm Gaussian kernel to improve the signal-to-noise ratio and the alignment between participant data (2 times the voxel size; Ref. 64), and *7*) wavelet despiked to remove any secondary motion artifacts (65) and *8*) had linear drifts removed (linear detrending due to scanner noise). Slice timing correction was not applied, following previous work on fast TR (subsecond) acquisition protocols (63). Data from four participants were excluded from further analysis because of head movement-related artifacts during the rs-fMRI acquisition, as measured by wavelet despiking [spike percentage higher than 10% (65)], resulting in a total of *N* = 18.

Next, we applied spatial group independent component analysis (ICA) using the Group ICA fMRI Toolbox (GIFT v3.0b) (http://mialab.mrn.org/software/gift/) to identify and remove components of noise. Principal component analysis was applied for dimensionality reduction, first at the subject level and then at the group level. The minimum description length criteria (66) were used to estimate the dimensionality and determine the number of independent components, resulting in 34 independent components. The ICA estimation (Infomax) was run 20 times, and the component stability was estimated with ICASSO (67). Following recent work on back-reconstruction methods for ICA denoising at the group level (68), we used group information guided ICA (GIG-ICA) back-reconstruction to reconstruct subject-specific components from the group components. We visually inspected the results and identified noise components according to published procedures (69). Using consensus voting among three experts (V. Karlaftis, P. Frangou, J. Giorgio), we labeled 11 of the 34 components as noise that captured signal from veins, arteries, CSF pulsation, susceptibility, and multiband artifacts.

To clean the fMRI signals from motion artifacts and the noise components, we followed a soft cleanup ICA denoise approach (70). That is, we first regressed out the motion parameters (translation, rotation, and their squares and derivatives; Ref. 71) from each voxel and ICA component time course. Second, we estimated the contribution of each ICA component to each voxel’s time course (multiple regression). Finally, we subtracted the unique contribution of the noise components from each voxel’s time course to avoid removing any shared signal between neuronal and noise components. We did not include the global signal as a nuisance regressor, as it has been shown to capture behaviorally relevant information (72) and neuronal signals (for review see Ref. 73). After ICA denoise, the data were high-pass filtered at 0.01 Hz and treated for serial correlations using the FAST autoregressive model (74, 75), and the residual time course from this step was used for all subsequent connectivity analyses.

#### Functional connectivity analysis.

We computed functional connectivity between the two MRS voxels. First, we computed the overlap across participant MRS voxels for EV and DLPFC separately and created group MRS masks that included voxels present in at least 50% of the participants’ MRS voxels. Then, for each participant and region of interest (ROI), we computed the first eigenvariate across all gray matter voxels within the ROI to derive a single representative time course per ROI.

We computed the functional connectivity between the EV and the DLPFC MRS voxels as the Pearson correlation between the eigenvariate time course from each of the MRS masks. We then applied Fisher *z*-transform to the correlation coefficient and averaged across runs to derive an EV-DLPFC connectivity value per participant. To test for specificity of the EV-DLPFC connectivity results, we computed the functional connectivity between EV and a control area [primary motor cortex (M1)]. We defined a left M1 mask of equal size to the MRS masks based on anatomical coordinates (MNI coordinates [−36, −26, 48]).

#### Statistical analysis.

To test for within-subject differences across measurements, we conducted a repeated-measures ANOVA in SPSS. For post hoc pairwise comparisons we tested for significance at *P* = 0.025 (Bonferroni corrected for 2 statistical tests). For testing the relationship of two or more variables, we used robust least-squares regression (*robustfit* function in MATLAB) for reweighting potential outliers. In particular, we used multiple regression models with two independent variables (DR and TH or Glu and GABA+) to minimize the number of statistical tests. Before performing a multiple regression, we ensured that the independent variables are not collinear. For all control analyses, we used a simple linear regression model with the variable of interest (i.e., the variable that showed a significant relationship) and tested for significant differences between predictors. For easier interpretation of the results, we also report a standardized *r* coefficient by converting the regression’s *t* statistic with the following formula: $r=sign(t)\times \sqrt{\frac{t^{2}}{t^{2}+df}}$. For visualization purposes, we plot the fitted lines according to the following formula: *y*_1,2_ = *b*_0_ + *b*_1,2_ ·*x*_1,2_ + *b*_2,1_ · mean(*x*_2,1_), where *y_i_* is the expected outcome value for the *i*th predictor, *b*_0_ is the beta weight for the constant term, *b_i_* is the weight for the *i*th predictor, and *x_i_* is the vector of the *i*th predictor. In line with previous MRS studies (76, 77), exploratory associations between additional functional connectivity measures (e.g., intrinsic connectivity) and our MRS and learning measures were assessed.

## RESULTS

### Training Alters Perceptual Decision Processes

We tested participants on an orientation identification task during a pretraining test block (without feedback) and five training blocks (with per-trial feedback) (Fig. 1*A*). On each trial, participants were asked to identify the orientation (45° or 135°) of a Gabor grating that was masked with Gaussian noise. Our results showed that participants improved in their judgments within a single training session (Fig. 1*B*), as indicated by significant differences in performance during training [repeated-measures ANOVA: main effect of block: *F*(5,105) = 3.04, *P* = 0.013]. In particular, following previous studies (38) using a single training session, we compared performance (accuracy) in the pretraining block to maximum training performance (max-training; i.e., performance at the training block with the higher accuracy between the last 2 training blocks per participant). Our results showed significantly higher performance after training [*t*(21) = 4.43, *P* < 0.001], consistent with previous reports showing behavioral improvement for early learning (i.e., within a single training session; for a review see Ref. 78).

We next asked whether training alters processes related to decision making. We modeled the data with five different drift diffusion models following previous work (11, 18). Using BIC as in previous studies (11, 12), we selected the model with the lowest mean BIC value across participants. We then extracted the following parameters related to decision processes from this model (*model 4*: DR-TH-Ter model): *1*) drift rate (DR), indicating the rate at which participants accumulate information for making a perceptual judgment, *2*) decision threshold (TH), indicating the amount of information required to make a judgment, and *3*) nondecision time (Ter), indicating the time for early encoding processes and late motor response processes. Comparing the model parameters between pretraining and max-training blocks, we found that drift rate significantly increased after training [*t*(21) = 4.48, *P* < 0.001; Fig. 1*C*] and decision threshold significantly decreased after training [*t*(21) = −3.85, *P* = 0.001; Fig. 1*D*], whereas no significant changes were observed for the nondecision time due to training [*t*(21) = 1.08, *P* = 0.293]. These results suggest that training improves the rate at which participants accumulate information and reduces the amount of evidence they require for making a decision, rather than non-decision-related processes.

### Glutamate Relates to Evidence Accumulation for Perceptual Decision Making

Recent work has linked visual cortex glutamatergic excitation and GABAergic inhibition to perceptual judgments and learning (for a review see Ref. 79). Here, we tested the role of excitatory (Glu) and inhibitory (GABA) neurotransmitters in perceptual decision making processes, as identified by diffusion modeling of performance in the orientation identification task. We measured Glu and GABA+ at rest (i.e., participants had their eyes open and fixated on a central cross) from voxels placed in *1*) the early visual cortex (EV MRS voxel; Fig. 2*A*), which is known to be involved in orientation discrimination and learning (24, 80), and *2*) the left dorsolateral prefrontal cortex (DLPFC MRS voxel; Fig. 2*A*), which is known to be involved in the readout of sensory information from visual cortex, transforming input to decision variables (81), and accumulating the decision variables during perceptual decision making (7, 8). Further, previous studies have shown that activity in DLPFC correlates with drift rate (7) and disruption of processing in left DLPFC with brain stimulation impairs performance accuracy, corresponding to decreased drift rate (8). To test the link between these neurotransmitters and learning-dependent changes in decision processes due to training on the orientation identification task, we related Glu and GABA+ levels in these regions with change (i.e., max-training block minus pretraining block) in the drift diffusion model parameters that showed significant differences due to training (multiple regression with DR and TH as independent variables).

We found a significant negative relationship between EV Glu and DR change after training but not TH change [multiple regression: DR: *b* = −2.13, *t*(19) = −2.83, *r* = −0.54, *P* = 0.011; TH: *b* = −0.41, *t*(19) = −0.24, *r* =−0.05, *P* = 0.815; Fig. 2*B*]. The relationship of EV Glu with DR change was significantly different from the relationship of EV Glu with TH change (*z* = −2.05, *P* = 0.041; EV Glu–DR: *r* = −0.59; EV Glu–TH: *r* = 0.09; DR–TH: *r* = −0.36), suggesting that EV Glu relates to DR rather than TH change. We did not observe any significant relationship between *1*) EV GABA+ and DR change or TH change [multiple regression: DR: *b* = −0.44, *t*(19) = −0.76, r = −0.17, *P* = 0.458; TH: *b* = −1.83, *t*(19) = −1.37, *r* = −0.30, *P* = 0.187; Fig. 2*C*)]; *2*) DLPFC Glu and DR change or TH change [multiple regression: DR: *b* = 1.04, *t*(14) = 1.17, *r* = 0.30, *P* = 0.262; TH: *b* = −4.46, *t*(14) = −1.10, *r* = −0.28, *P* = 0.291; Fig. 2*D*]; and *3*) DLPFC GABA+ and DR change or TH change [multiple regression: DR: *b* = −0.02, *t*(14) = −0.04, *r* = −0.01, *P* = 0.969; TH: *b* = −3.17, *t*(14) = −1.54, *r* = −0.38, *P* = 0.146; Fig. 2*E*]. The relationship between EV Glu and DR change remained significant when we performed the following control analyses: *1*) referenced Glu to NAA rather than water [*b* = −1.71, *t*(20) = −3.11, *r* = −0.57, *P* = 0.006]; *2*) excluded four participants because of poor Gln fit [*b* = −2.47, *t*(16) = −3.51, *r* = −0.66, *P* = 0.003]; and *3*) controlled for MRS data quality [absolute CRLB: *b* = −2.58, *t*(20) = −4.75, *r* = −0.73, *P* < 0.001; linewidth: *b* = −1.78, *t*(20) = −3.00, *r* = −0.56, *P* = 0.007; SNR: *b* = −2.27, *t*(20) = −3.97, *r* = −0.66, *P* = 0.001]. Further, the relationship of EV Glu with DR change was significantly different from the relationship of EV GABA+ with DR change (*z* = −2.07, *P* = 0.038; EV Glu–DR: *r* = −0.59; EV GABA+–DR: *r* = −0.09; EV Glu–GABA+: *r* = 0.26), suggesting that EV Glu rather than GABA+ relates to information accumulation. There was no significant relationship between EV Glu and DR before training [*b* = 0.94, *t*(20) = 1.01, *r* = 0.22, *P* = 0.324], suggesting that our results could not be simply due to variability in pretraining performance. These results indicate a significant contribution of DR change to EV Glu, suggesting that faster rates of information accumulation after training relate to lower glutamatergic excitation in early visual cortex.

### Visual-DLPFC Functional Connectivity for Perceptual Decision Making

Previous work has shown that functional connectivity at rest predicts individual variability in a range of tasks (for reviews see Refs. 82, 83), including perceptual learning (38, 84). Further, previous studies have linked functional connectivity in visual and frontal cortex to perceptual judgments and learning-dependent plasticity (for reviews see Refs. 85, 86). Here, we tested whether functional interactions between early visual cortex and DLPFC, as measured by rs-fMRI, relate to decision making processes and neurochemical processing (glutamatergic, GABAergic) when training on an orientation identification task.

**Figure 3.**
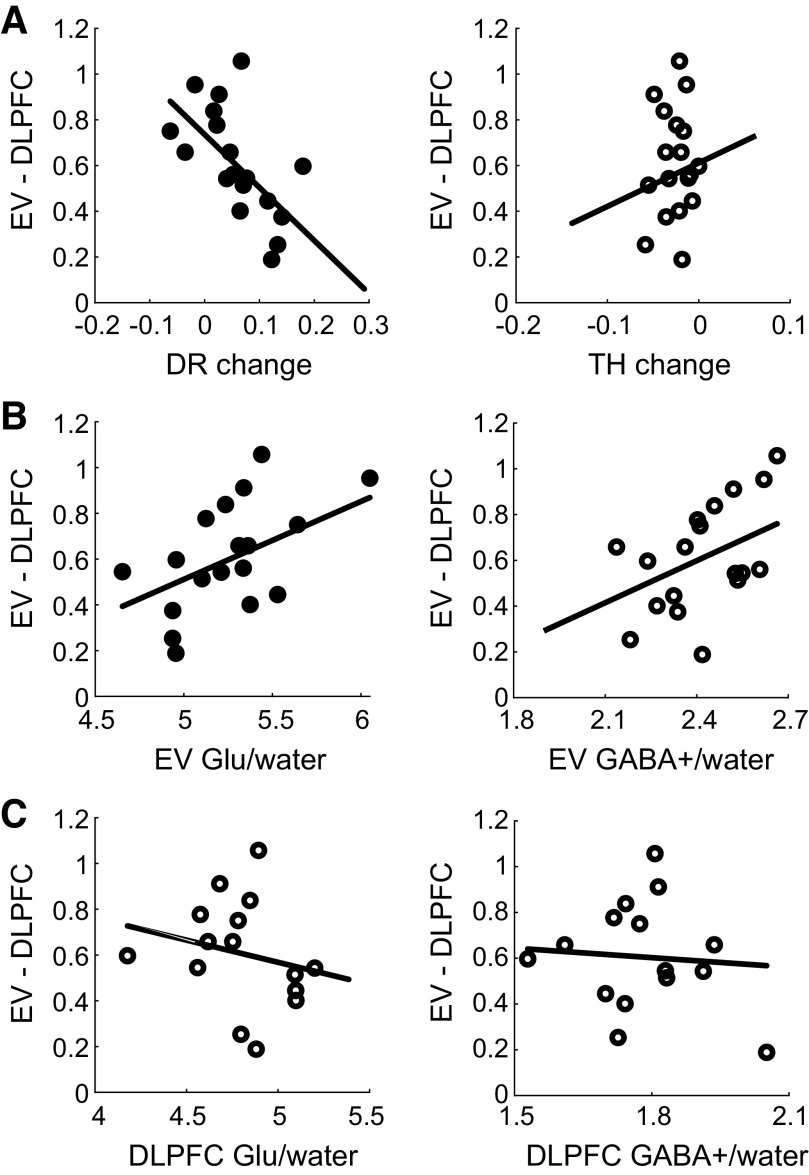
Relationship of early visual cortex (EV)-dorsolateral prefrontal cortex (DLPFC) functional connectivity to behavior and glutamate (Glu). EV-DLPFC functional connectivity (Fisher’s *z*), as measured by resting-state functional magnetic resonance imaging (rs-fMRI), shows a significant negative linear relationship with drift rate (DR) but not decision threshold (TH) change (multiple regression) (*A*), a significant positive linear relationship with EV Glu but not EV GABA+ (*B*), and no significant linear relationship with DLPFC Glu or GABA+ (*C*). Significant results are indicated by filled symbols and nonsignificant results by open symbols.

First, we tested whether functional connectivity between EV and DLPFC relates to drift rate and decision threshold (multiple regression with DR and TH as independent variables). We measured functional connectivity as the correlation between rs-fMRI time courses from gray matter voxels within the EV and DLPFC voxels (EV-DLPFC connectivity). We observed a significant negative relationship between EV-DLPFC functional connectivity and DR change but not TH change [multiple regression: DR: *b* = −2.32, *t*(15) = −2.94, *r* = −0.60, *P* = 0.010; TH: *b* = 1.91, *t*(15) = 0.63, *r* = 0.16, *P* = 0.538; Fig. 3*A*). The relationship of EV-DLPFC functional connectivity with DR change was significantly different from the relationship of EV-DLPFC functional connectivity with TH change (*z* = −2.03, *P* = 0.043; EV-DLPFC connectivity–DR: *r* = −0.60; EV-DLPFC connectivity–TH: *r* = 0.07; DR–TH: *r* = −0.36), suggesting that EV-DLPFC functional connectivity relates to DR rather than TH change. There was no significant relationship between EV-DLPFC functional connectivity and DR before training [*b* = 0.59, *t*(16) = 0.66, *r* = 0.16, *P* = 0.521], suggesting that our results could not be simply due to variability in pretraining performance. We did not observe a significant relationship of functional connectivity between early visual cortex and a control region (M1) with DR change [*b* = −1.57, *t*(16) = −1.64, *r* = −0.38, *P* = 0.121] or when controlling for the relationship with EV-DLPFC connectivity [*b* = 0.41, *t*(16) = 0.31, *r* = 0.08, *P* = 0.762], suggesting that our results are specific to EV-DLPFC connectivity. Thus, our results indicate a significant contribution of DR change to EV-DLPFC connectivity, suggesting that faster rates of information accumulation due to training relate to lower functional connectivity between early visual and dorsolateral prefrontal cortex.

Second, we tested whether EV-DLPFC functional connectivity relates to glutamatergic or GABAergic processing in EV and DLPFC (multiple regression with Glu and GABA+ as independent variables). We observed a significant positive relationship between EV-DLPFC connectivity and EV Glu but not EV GABA+ [multiple regression: EV Glu: *b* = 0.34, *t*(15) = 2.18, *r* = 0.49, *P* = 0.046; EV GABA+: *b* = 0.61, *t*(15) = 1.89, *r* = 0.44, *P* = 0.078; Fig. 3*B*). The relationship of EV-DLPFC functional connectivity with EV Glu was not significantly different from that of EV-DLPFC functional connectivity with EV GABA+ (*z* = 0.22, *P* = 0.824; EV-DLPFC connectivity–EV Glu: *r* = 0.53; EV-DLPFC connectivity–EV GABA+: *r* = 0.48; EV Glu–EV GABA+: *r* = 0.26). We did not observe any significant relationships between EV-DLPFC and DLPFC Glu or DLPFC GABA+ [multiple regression: DLPFC Glu: *b* = −0.19, *t*(12) = −0.64, *r* = −0.18, *P* = 0.531; DLPFC GABA+: *b* = −0.14, *t*(12) = −0.22, *r* = −0.06, *P* = 0.826; Fig. 3*C*). The relationship between EV-DLPFC connectivity and EV Glu remained significant when we performed the following control analyses: *1*) referenced Glu to NAA rather than water [*b* = 0.66, *t*(16) = 2.88, *r* = 0.58, *P* = 0.011]; *2*) excluded four participants because of poor Gln fit [*b* = 0.51, *t*(13) = 2.87, *r* = 0.62, *P* = 0.013]; and *3*) controlled for MRS data quality [absolute CRLB: *b* = 0.55, *t*(16) = 3.07, *r* = 0.61, *P* = 0.007; linewidth: *b* = 0.42, *t*(16) = 2.56, *r* = 0.54, *P* = 0.021; SNR: *b* = 0.44, *t*(16) = 2.74, *r* = 0.57, *P* = 0.015]. Further, we found no significant relationship between EV-M1 functional connectivity and EV Glu [*b* = 0.33, *t*(16) = 1.72, *r* = 0.40, *P* = 0.104] or when controlling for the relationship with EV-DLPFC connectivity [*b* = −0.08, *t*(16) = −0.33, *r* = −0.08, *P* = 0.744], suggesting that our results are specific to EV-DLPFC connectivity. Thus, our results indicate a significant contribution of EV Glu to EV-DLPFC connectivity, suggesting that lower early visual cortex excitation relates to lower functional connectivity between early visual and dorsolateral prefrontal cortex to support faster rates of information accumulation.

### Increasing Excitation in the Visual Cortex Impairs Learning

To extend beyond correlational approaches, we employed anodal tDCS to perturb cortical excitability during training on the orientation identification task. Anodal tDCS is an excitatory stimulation protocol that has been shown to increase cortical excitability in visual (87) and motor (88) cortex. We have previously shown that anodal tDCS results in improved learning in the context of a visual task that requires enhanced excitability (37). As our main experiment showed that lower visual cortex excitation relates to faster drift rate after training on the orientation identification task, we hypothesized that excitatory stimulation would impair learning compared with sham stimulation.

**Figure 4.**
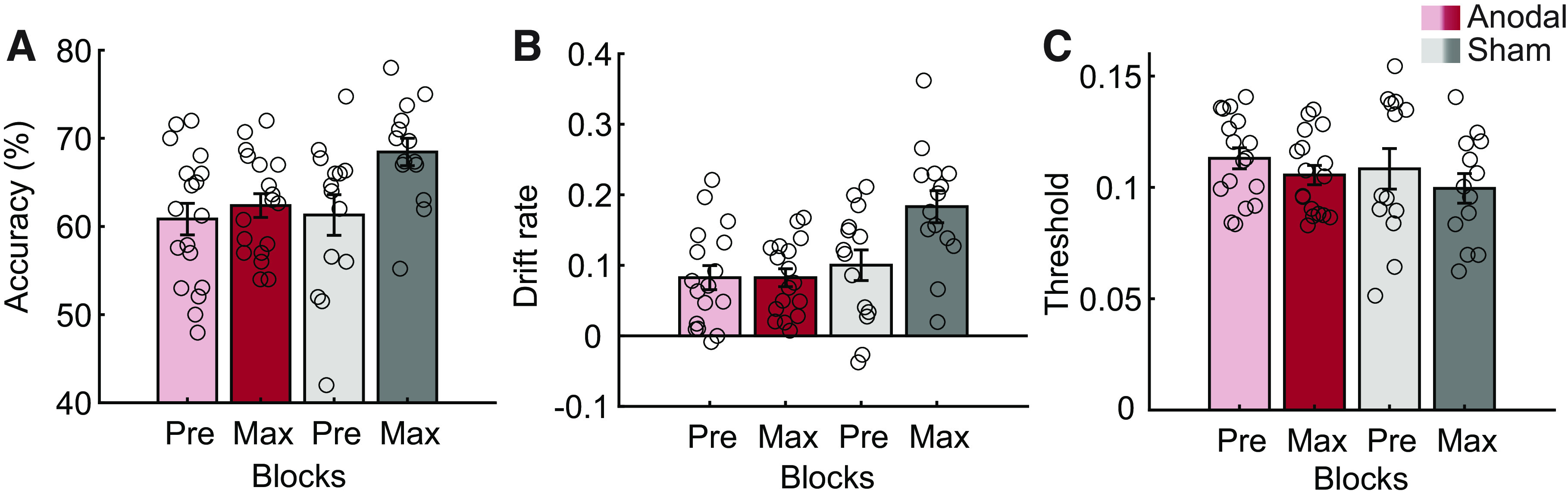
Transcranial direct current stimulation (tDCS) intervention: mean accuracy (*A*), drift rate (*B*), and decision threshold (*C*) across participants in the Anodal and Sham groups for the pretest (Pre) and max-training (Max) blocks. Error bars indicate SE across participants. Open circles indicate individual participant data.

To test this hypothesis, we trained two groups of participants on the orientation identification task, one receiving anodal and the other sham stimulation during training. As in the main experiment, we compared accuracy, DR, and TH in the max-training block against the pretraining block. We found that participants who received anodal stimulation during training showed lower improvement after training compared with those who received sham stimulation. In particular, a repeated-measures ANOVA on accuracy showed a significant Group (Anodal, Sham) × Block (pretraining, max-training) interaction [*F*(1,30) = 4.68, *P* = 0.039; Fig. 4*A*] and post hoc comparisons showed significant performance improvement after training (i.e., increased accuracy) for the Sham [*t*(13) = 3.23, *P* = 0.004] but not the Anodal [*t*(17) = 0.95, *P* = 0.356] group.

Further, a repeated-measures ANOVA on DR showed a significant Group (Anodal, Sham) × Block (pretraining, max-training) interaction [*F*(1,29) = 8.39, *P* = 0.007; Fig. 4*B*]. Post hoc comparisons showed significant changes in DR after training (i.e., faster drift rate) for the Sham [*t*(13) = 3.07, *P* = 0.009] but not the Anodal [*t*(16) = −0.001, *P* = 0.999] group, suggesting that participants in the Anodal group showed slower drift rate after training compared with those in the Sham group. Finally, a repeated-measures ANOVA on TH showed a significant main effect of Block [*F*(1,29) = 10.59, *P* = 0.003; Fig. 4*C*] but no significant Group (Anodal, Sham) × Block (pretraining, max-training) interaction [*F*(1,29) = 0.88, *P* = 0.356], suggesting that the effect of the anodal stimulation was specific to the rate of information accumulation. Note that DR before training was not different between the Anodal and Sham groups [Anodal vs. Sham: *t*(29) = 0.65, *P* = 0.521] and did not differ between the stimulation groups and the main study [1-way ANOVA with Group (Anodal, Sham, no stimulation): *F*(2,52) = 0.57, *P* = 0.571], suggesting that the tDCS effects we observed after training were not due to variability across participants before training. We found similar results in a smaller group of participants (after removing 6 participants from the Anodal group who performed the task at a lower contrast level; see materials and methods; Supplemental Fig. S1); that is, repeated-measures ANOVAs showed a significant Group × Block interaction for DR [*F*(1,24) = 5.20, *P* = 0.032, post hoc for Anodal: *t*(11) = 1.09, *P* = 0.301] but not TH [*F*(1,24) = 0.27, *P* = 0.610].

## DISCUSSION

Training is known to improve perceptual decisions. Here, we tested the neurochemical and functional connectivity mechanisms that support improved perceptual decisions due to training. Using MRS, we tested for glutamatergic and GABAergic processing in early visual and decision-related regions. Using rs-fMRI, we tested for functional interactions between these regions that relate to decision processes. Modeling behavioral performance with a drift diffusion model, we demonstrate that training results in faster evidence accumulation for orientation identification. These learning-dependent changes in decision processes relate to glutamate levels in visual cortex and functional connectivity between visual and dorsolateral prefrontal cortex. Our results suggest that efficient sensory processing and functional interactions between sensory and decision-related regions support improved decision making through training. Further, perturbing cortical excitability with tDCS disrupts evidence accumulation during training, providing a direct link between visual cortex excitation and perceptual decisions. Our findings advance our understanding of the role of learning in decision making in the following respects.

First, we show that training improves behavioral performance on a visual orientation identification task by increasing the information accumulation rate and reducing the information needed to make a judgment. This is consistent with previous studies showing that training facilitates information accumulation for perceptual decision making (11, 12, 16, 18). Further, our results using single-session training are consistent with previous work showing learning-dependent changes early in the training (37, 38, 89).

Second, we demonstrate that glutamatergic excitation in the early visual cortex relates to early learning-dependent changes in sensory information processing during the decision processes (4, 14). Our results show that lower resting levels of early visual cortex glutamate, rather than GABA+, relate to increased drift rate after training, suggesting that lower excitatory processing in visual cortex relates to faster information accumulation after training. This relationship is shown to be specific to glutamatergic rather than GABAergic processing in visual cortex. Although it remains debated whether MRS measures synaptic versus extrasynaptic neurotransmitter concentration (90), some previous studies have linked glutamatergic excitation to visual discriminations (e.g., Refs. 31–33) and others GABAergic inhibition to performance in visual tasks (34, 37–39, 91, 92). Our results provide evidence that cortical glutamatergic excitation, known to relate to gain control mechanisms (93), is involved in information accumulation during decision making.

Previous studies have implicated frontoparietal networks in information accumulation during visual tasks (94–96); yet recent evidence suggests that stimulus (rather than value) information accumulation engages visual areas (97). Our results highlight a key role for early visual cortex in decision making processes, showing that glutamate in early visual cortex (as measured by MRS at rest) relates to increased accumulation of information after training. This relationship was not significant for drift rate before training, suggesting a link between excitatory processing in visual cortex and improved perceptual decisions after training. It is possible that optimizing information accumulation with training relates to more efficient input processing in the visual cortex that involves reduced excitatory processing. This interpretation is consistent with previous studies showing that lower functional MRI (fMRI) signal in decision-related areas relates to shorter duration of information accumulation (96).

Further, it is possible that in the presence of external noise training reduces activity in visual cortex, as reflected by lower levels of glutamatergic excitability and reduced learning under excitatory stimulation. These reduced levels of excitation may correspond to exclusion of external noise (98), resulting in improved behavioral performance at early stages of learning (i.e., the single training protocol employed in our study). The lack of a significant relationship between DLPFC MRS measures and learning may suggest that learning, at early stages of training (i.e., single training session), alters stimulus processing (i.e., sensory processes) in early visual cortex rather than information accumulation processes in DLPFC. These results are consistent with the reverse hierarchy theory of perceptual learning, suggesting that training on difficult tasks (as in the case of the task employed in our study) engages early visual cortex (99).

Extending beyond correlational approaches, our tDCS intervention provides evidence for a direct link between excitatory processing in visual cortex and perceptual decisions, showing that increasing levels of excitation in the visual cortex through anodal tDCS disrupts information accumulation during training. At first glance, our results appear to be in contrast to previous studies showing that anodal tDCS facilitates performance in visual perception and memory tasks that involve excitatory processing (37, 100). Yet the disruption of learning we observed due to anodal tDCS is in agreement with the negative relationship between visual cortex excitation and rate of information accumulation in the context of our orientation identification task. Interestingly, previous work using transcranial random noise stimulation (tRNS) during training on a fine orientation discrimination task has shown that tRNS improves performance compared to anodal or sham tDCS (101, 102). Although its mechanism of action remains debated, it is proposed that tRNS boosts signal detection by introducing stochastic resonance and enhancing processing of subthreshold stimuli (103). As low-contrast signal detection (103) and information accumulation in a perceptual decision making task (104) have been shown to benefit from tRNS, it would be interesting to test in future studies whether tRNS stimulation improves orientation identification performance.

Third, we demonstrate that functional connectivity between visual and decision-related regions relates to learning-dependent changes in decision making processes and glutamatergic processing in visual cortex. In particular, our results show that lower visual-frontal connectivity relates to faster information accumulation due to training and lower excitatory input processing, as indicated by lower levels of glutamate in visual cortex. It is possible that faster information accumulation due to training relates to more efficient local processing in visual cortex and interactions between visual and decision-related regions. This is consistent with previous work implicating local gain control mechanisms in visual cortex and reduced interareal connectivity when learning to identify targets in noise (38). Further, our findings highlight the role of neurochemical mechanisms in network connectivity, consistent with previous studies showing a link between glutamate levels and functional connectivity at rest within and between brain regions (42, 43).

In sum, our findings provide novel insights in understanding the neurochemical mechanisms that underlie perceptual decision making. Combining multimodal brain imaging (MRS, rs-fMRI) with brain stimulation and computational modeling reveals a key role of glutamatergic processing for perceptual decisions. Our findings demonstrate that efficient local processing related to glutamatergic excitation and interareal connectivity supports improved perceptual decisions through training. In this work, we focused on measurements of neurotransmitters and connectivity at rest. Future work combining tDCS with multimodal brain imaging during training could investigate functional changes in neurotransmission to uncover its role in regulating network activity and connectivity for learning and brain plasticity.

## DATA AVAILABILITY

The data that support the findings of this study are openly available https://doi.org/10.17863/CAM.82236.

## SUPPLEMENTAL DATA

10.17863/CAM.82236 Supplemental Fig. S1 and Tables S1 and S2: https://doi.org/10.17863/CAM.82236.

## GRANTS

This work was supported by grants to Z.K. from the Biotechnology and Biological Sciences Research Council (grant numbers H012508, BB/P021255/1), the Wellcome Trust (grant number 205067/Z/16/Z), and the European Community’s Seventh Framework Program (FP7/2007–2013) under agreement PITN-GA-2011-290011 and to K.J. from the European Union’s Horizon 2020 research and innovation program under grant agreements number 840271.

## DISCLOSURES

No conflicts of interest, financial or otherwise, are declared by the authors.

## AUTHOR CONTRIBUTIONS

K.J., P.F., and Z.K. conceived and designed research; K.J., P.F., J.G., R.R., E.Z., and V.H. performed experiments; K.J., P.F., V.M.K., J.J.Z., V.H., and U.E. analyzed data; K.J., P.F., V.M.K., J.J.Z., and Z.K. interpreted results of experiments; K.J., P.F., V.M.K., and J.J.Z. prepared figures; K.J., P.F., V.M.K., J.J.Z., and Z.K. drafted manuscript; K.J., P.F., V.M.K., J.J.Z., J.G., R.R., E.Z., V.H., U.E., and Z.K. edited and revised manuscript; K.J., P.F., V.M.K., J.J.Z., J.G., R.R., E.Z., V.H., U.E., and Z.K. approved final version of manuscript.

## ENDNOTE

At the request of the authors, readers are herein alerted to the fact that additional materials related to this manuscript may be found at https://doi.org/10.17863/CAM.82236. These materials are not a part of this manuscript and have not undergone peer review by the American Physiological Society (APS). APS and the journal editors take no responsibility for these materials, for the website address, or for any links to or from it.
